# Estimating the correlation between rates of change in longitudinal studies in presence of dropout

**DOI:** 10.64898/2026.10.16.26365737

**Published:** 2026-10-20

**Authors:** Ruben P. Dörfel, Brice Ozenne, the Alzheimer’s Disease Neuroimaging Initiative (ADNI)

## Abstract

Estimating the correlation between subject-specific rates of change is a common objective in longitudinal studies. A natural approach is to fit ordinary least squares regressions per subject and correlate the resulting slope estimates directly. Under complete data, this slopes-first estimator is algebraically equivalent to a contrast-based plug-in estimator derived from the joint variance-covariance matrix of the two signals across time. Under independent dropout, however, the slopes-first approach implicitly applies heterogeneous weights across missing-data patterns, introducing bias whenever the cross-covariance between the two signals is not constant over time. We formalise this result and propose an unbiased alternative that estimates the joint covariance matrix before applying the contrast. We further extend our results to include covariates as well. Simulations confirm that the slopes-first estimator is biased under non-constant cross-covariance while the proposed alternative remains unbiased. Further, we show in a real-world example that the two estimators can lead to different scientific conclusions and recommend that researchers working with longitudinal data inspect the missing data pattern to make an informed decision about the estimator of choice.

